# Seasonal and Spatial Environmental Influence on *Opisthorchis viverrini* Intermediate Hosts, Abundance, and Distribution: Insights on Transmission Dynamics and Sustainable Control

**DOI:** 10.1371/journal.pntd.0005121

**Published:** 2016-11-23

**Authors:** Christina Sunyoung Kim, Pierre Echaubard, Apiporn Suwannatrai, Sasithorn Kaewkes, Bruce A. Wilcox, Banchob Sripa

**Affiliations:** 1 WHO Collaborating Centre for Research and Control of Opisthorchiasis (Southeast Asian Liver Fluke Disease), Tropical Disease Research Laboratory, Department of Pathology, Faculty of Medicine, Khon Kaen University, Khon Kaen, Thailand; 2 Department of Biology, Laurentian University, Sudbury, Ontario, Canada; 3 Global Health Asia, Faculty of Public Health, Mahidol University, Bangkok, Thailand; 4 Department of Parasitology, Faculty of Medicine, Khon Kaen University, Khon Kaen, Thailand; Vienna, AUSTRIA

## Abstract

**Background:**

*Opisthorchis viverrini* (*Ov*) is a complex-life-cycle trematode affecting 10 million people in SEA (Southeast Asia). Human infection occurs when infected cyprinid fish are consumed raw or undercooked. *Ov* requires three hosts and presents two free-living parasitic stages. As a consequence *Ov* transmission and infection in intermediate and human hosts are strongly mediated by environmental factors and understanding how environmental variability influences intermediate host abundance is critical. The objectives of this study were 1) to document water parameters, intermediate hosts abundance and infection spatio-temporal variation, 2) to assess their causal relationships and identify windows of transmission risk.

**Methodology/Principal Findings:**

Fish and snails were collected monthly for one year at 12 sites in Lawa Lake, an *Ov-*endemic region of Khon Kaen Province in Northeast Thailand. Physicochemical water parameters [pH, temperature (Tp), dissolved oxygen (DO), Salinity, electrical conductivity (EC), total dissolved solid (TDS), nitrite nitrogen (NO_2_-N), lead (Pb), total coliform bacteria (TCB) and fecal coliform bacteria (FCB)] were measured. Multivariate analyses, linear models and kriging were used to characterize water parameter variation and its influence on host abundance and infection prevalence. We found that sampling sites could be grouped in three clusters and discriminated along a nitrogen-salinity gradient where higher levels in the lake’s southern region predicted higher *Bithynia* relative abundance (*P*<0.05) and lower snail and fish species diversity (*P*<0.05). Highest *Bithynia* abundance occurred during rainy season (*P*<0.001), independently of site influence. Cyprinids were the most abundant fish family and higher cyprinid relative abundance was found in areas with higher *Bithynia* relative abundance (*P*<0.05). *Ov* infection in snails was anecdotal while *Ov* infection in fish was higher in the southern region (*P*<0.001) at sites showing high FCB.

**Conclusions/Significance:**

Our results indicate that water contamination and waterways configuration can influence freshwater communities’ assemblages possibly creating ideal conditions for sustained transmission. Sustainable control may require a better appreciation of the system’s ecology with wise governance and development planning particularly in the current context of SEA agricultural intensification and landscape modification.

## Author Summary

*Opisthorchis viverrini* (*Ov*) is a fish-borne parasite infecting humans when they consume raw or undercooked fish. *Ov* is endemic in Southeast Asia, particularly in rural parts of Northeast Thailand, Lao PDR, Cambodia and Vietnam. The *Ov* lifecycle includes three different hosts: snails, fish and humans. Transmission takes place in various environments and water habitats where many factors interact and influence *Ov* survival and transmissibility from host to host. Our study investigated the influences of water parameters on snail and fish abundance and *Ov* infection rates. We found that salinity and nitrite-nitrogen were positively correlated to *Bithynia* snail relative abundance and inversely correlated to snail and fish species diversity. Cyprinids were the most abundant fish family and high cyprinid abundance was found in areas with high *Bithynia* abundance creating ideal conditions for transmission. *Ov* infection in fish was consistently detected from an area with high levels of salinity and nitrite-nitrogen and characterized with high abundance of *Bithynia* and cyprinids. Our findings suggest that water contamination can influence freshwater communities’ assemblages possibly creating ideal conditions for sustained parasite transmission. Sustainable *Ov* control may require a better appreciation of ecological systems together with wiser governance and development planning.

## Introduction

*Opisthorchis viverrini* (*Ov*), the Southeast Asian liver fluke, is a fish-borne complex life cycle trematode endemic in Thailand, Lao PDR, Cambodia and southern parts of Vietnam where an under-estimate of 10 million people are reported to be at risk of *Ov* infection [1, 2]. While most infections are asymptomatic, heavy chronic infections are associated with clinical hepatobiliary complications such as cholangitis, advanced periductal fibrosis, hepatomegaly and in some rare cases cholangiocarcinoma, a bile duct cancer associated with very poor prognosis upon diagnosis [1, 3, 4]. The northeast region of Thailand is known in particular to be a hotspot of *Ov* endemicity which despite nationwide public health prevention campaigns led by the government and private organizations [5], still is plagued with high infection prevalence [3, 6]. The persistence of high infection rate in the region is likely due to its cultural and ecological particularities where wet rice agrarian habitats; centuries old raw food culture and the parasite complex biology combine to create an ideal transmission arena [3, 7].

The parasite complex lifecycle begins when *Ov* eggs are released in the environment through the feces of a definitive human host or reservoir host, which are mostly cats and dogs [8]. Upon reaching freshwater habitats, *Ov* eggs are eventually ingested by freshwater snails belonging to the family Bithyniidae. Within the snail, *Ov* eggs hatch and the emerging miracidia develop to become sporocysts, which undergo asexual multiplication. The sporocysts develop to rediae and then finally to their free-swimming cercaria stage that will be released in the environment. Thousands of cercariae can be released as free-swimming parasites into the aquatic environment where they actively search for certain species of freshwater fish of the Cyprinidae family, the second intermediate host. Upon contact with the fish, cercariae encyst within the fish body and develop into infective metacercariae. *Ov* metacercariae can infect humans when the fish that contained them are consumed raw or not cooked sufficiently to alter the parasite’s infectious potential [9].

Research conducted in the fields of immunology and pathology has greatly improved our ability to punctually diagnose, treat and respond to *Ov* and other liver fluke infections [10–12]. However, there is a lack of robust understanding of the ecological and environmental determinants of *Ov* transmission and therefore strong limitations remain regarding the sustainable interruption of the transmission and effective control. The inherent complexity of the *Ov* lifecycle, including the need of three taxonomically different hosts with markedly different ecologies, provides ample opportunity for environmental modification at different spatial and temporal scales to modulate patterns of transmission [13].

Biotic factors such as toxic exudates produced by hosts, non-hosts, predators and decoy organisms may act simultaneously and in conjunction with abiotic factors to expose free-living endohelminth stages to a complex array of hazards on their way to the down-stream host [14]. Similarly water-related environmental parameters can strongly influence host physiological status [15], demography [16] and distribution and modulate patterns of host-parasite encounter, hence transmission dynamics and infection likelihood. For example natural environmental variables such as temperature, salinity and pH have strong species- and stage-specific effects on survival rates [17]. In the case of *Ov*, infection likelihood in snails has been recently shown to be temperature-dependent [18]. Environmental disturbances, which can affect freshwater snail community structure, including species diversity and relative abundance [19, 20] contribute to modulate parasite transmission. For example, more species-diverse snail communities cause a 25–50% reduction in infection among *Schistosoma mansoni* snail hosts (*Biomphalaria glabrata*) and infected snails raised alongside non-host snails (*Lymnaea* or *Helisoma* sp.) also produce 60–80% fewer cercaria, suggesting that diverse snail communities could reduce human infection risk and that environmental change impacting host ecological functioning are important overarching determinants that modulate transmission [21, 22].

In the context of the ongoing agricultural intensification, landscape modification, and livelihood changes in rural SEA [23], wetland water contamination is increasing, natural biogeochemical cycles are disrupted, natural and human hosts demography are remodeled and as a consequence infectious disease risk in general and *Ov* incidence in particular fluctuates spatially and temporally. A dynamic view acknowledging the spatial and temporal interactions between environmental variability, host distribution and abundance and their consequence on transmission and infection at multiple scales is thus critical to improve our ability to identify pathogenic landscapes and refine our intervention strategies [24]. This rationale is particularly relevant in the case of *Ov*, which transmission interruption implies understanding cultural behaviors, socio-economic shifts and environmental particularities [25, 26].

The objective of this study was two-fold: 1) to document water parameters, intermediate hosts abundance and *Ov* infection spatial and seasonal variation, 2) to assess their causal relationships and identify windows of transmission risk in an *Ov* endemic ecosystem characterized by high rate of environmental and livelihood changes.

## Materials and methods

### Study sites

**Fig 1:**
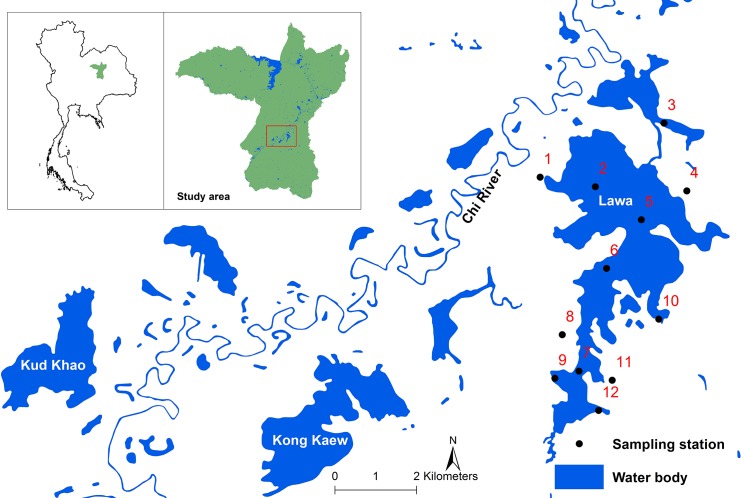
Map of the 12 sampling sites in Lawa Lake, which is approximately located in Ban Phai District of Khon Kaen Province, Northeast Thailand.

Khon Kaen Province, in Northeast Thailand, has been acknowledged as an area of high endemicity and ongoing *Ov* infection with certain wetland areas characterized by particularly high infection prevalence. For instance the Kaeng Lawa Reservoir, more commonly referred to as Lawa Lake, located in Ban Phai District, is known for its high and persistent endemicity of *Ov* infection [6]. Conversations with local people in the area have indicated that communities are strongly relying on the lake and its tributaries for subsistence. As a result fish preparations, including raw/fermented/undercooked fish dishes, are particularly frequently consumed and appreciated. Rice cultivation is also a dominant activity in the area, which not only provides an ideal habitat for snails, the first intermediate host, but also fosters the prolonged presence of local farmers in the environment and a high likelihood for open defecation, hence sustained parasite transmission. As a consequence Lawa Lake is a suspected important focal point of transmission, but known local differences in lake geomorphology and seasonal variability in water movements may imply heterogeneous transmission risk among lakeshore localities [27, 28]. Khon Kaen Province has a tropical monsoon climate with three major seasons: hot-dry (March-June), hot-rainy (July-October) and cool-dry (November-February) seasons. The rainy season comprises severe weather events such as heavy rainfall, floods and sometimes even drought that can strongly influence humans and wildlife through seasonal habitats modification. For instance floods in Lawa Lake typically occur during the months of September and October. During this time the water level of the Chi River will rise so high that some areas on the west side of the lake such as in Ban Chikokkor (Fig 1), will be completely submerged forcing local communities to use boats to commute from place to place. These important seasonal variations in water level are likely to strongly influence *Ov* eggs movement from latrines to the environment and snail and fish distribution and abundance, hence *Ov* transmission dynamics. In 2013, during which our research was implemented, temperatures in Thailand were higher than usual from January to November and lower than usual in December. Thailand was hit by a tropical storm in October that was downgraded from typhoon Wutip [29]. These storms also brought unusual rainfall in November and December and contributed to the lower than normal temperatures that occurred in Northeast Thailand [29].

Twelve sampling sites within the main body and near the shore of Lawa Lake were selected for the systematic collection of *Bithynia* snails and cyprinid fish and to perform a water quality assessment over a 1-year study period. The number and location of sampling sites were chosen to provide a representation of the main aquatic habitats found in and around the lake and therefore to account for their influence on water quality variability. Among the 12 sites, 8 were located 5 meters (m) from the shore and categorized as ‘nearshore sites’ and described based on their immediate surroundings to various types of rich and aquatic vegetation. This included: ‘shallow grassland,’ indicating a water depth ≤2 m; and ‘deeper grassland’ indicating a water depth >3 m (see Table 1). ‘Nearshore sites’ are exposed to desiccation and strong water quality fluctuation. An additional four ‘offshore sites’ were selected to account for the deepest areas of Lawa Lake. The ‘offshore sites’ contrast with ‘nearshore sites’ in their tendency to remain submerged even during the hot dry season creating refuges for fish during the summer months. Nearshore sites in particular were chosen to be in close proximity to villages for which *Ov* infection prevalence data from the Tropical Disease Research Laboratory, Khon Kaen University [6] is available. The investigation of the snail and fish hosts’ seasonal abundance and infection trends has been intended to improve our understanding of *Ov* prevalence and infection intensity variation in their neighboring local communities.

**Table 1:** Geospatial characteristics of the 12 sampling sites in Lawa Lake selected for the study.

ID	Zone	Category	Description	Village	Infection rate^a^	Latitude	Longitude
1	Upper	Near shore	Shallow grassland	Chikokkor	55%	16°9'38.09"N	102°39'51.98"E
2	Upper	Off shore	Deep	-	-	16° 9'30.51"N	102°40'35.75"E
3	Upper	Near shore	Shallow grassland	Khoksamran	15%	16°10'20.93"N	102°41'30.16"E
4	Upper	Near shore	Shallow grassland	Donpordang	18%	16° 9'27.21"N	102°41'48.15"E
5	Upper	Off shore	Deep	-	-	16° 9'4.34"N	102°41'12.20"E
6	Upper	Off shore	Deep	-	-	16° 8'25.81"N	102°40'44.63"E
7	Lower	Off shore	Deep	-	-	16° 7'4.36"N	102°40'22.75"E
8	Lower	Near shore	Deeper grassland	-	-	16° 7'33.30"N	102°40'9.53"E
9	Lower	Near shore	Deeper grassland	-	-	16° 6'58.71"N	102°40'3.64"E
10	Lower	Near shore	Shallow grassland	Tad	65%	16° 7'45.59"N	102°41'25.96"E
11	Lower	Near shore	Shallow grassland	Pao	65%	16° 6'57.15"N	102°40'49.04"E
12	Lower	Near shore	Deeper grassland	Nongnangkwan	74%	16° 6'33.22"N	102°40'38.27"E

^a^ *Ov* infection data taken from Sripa et al., 2015

### Field timeline and sample collection

Samples were collected on the third week of every month from February 2013 to January 2014. This was to create consistency and allow a week of time each month as a safety-net-period in case samples could not be collected due to outside circumstances. Data collected at the 12 sites included 10 water parameters as well as cyprinid fish. Snails were collected from 8 out of the 12 sites since they are not present in water depths exceeding 3 meters [30]. A portable Global Positional System (GPS) unit was used to record the latitude and longitude coordinates at each sampling location.

### Water sampling and testing

Five physicochemical water parameters were measured *in situ* 30 cm below the surface of the water from a steady boat to reduce sediment disturbance using a portable water meter (Extech Oyster DO 0700 Meter) that was calibrated according to instrument guidelines. The parameters measured by the Extech meter included temperature, pH, electrical conductivity (EC), total dissolved solid (TDS) and salinity. Additionally, water samples were collected in sterilized 500 mL sized polyethylene and 100 mL glass bottles and transported on ice to the Environmental Laboratory of Regional Environment Office 10 Khon Kaen for analysis. The parameters measured there were dissolved oxygen (DO), nitrite nitrogen (NO_2_-N), lead (Pb), total coliform bacteria (TCB) and fecal coliform bacteria (FCB). Levels of DO were measured using the Azide Modification Method; Pb, by the Nitric digestion method, GFAAS; NO_2_-N, by the colorimetric method; and TCB and FCB were both tested using the most probable number (MPN) technique per 100 mL sample of water.

### Snail sampling and cercarial shedding

Snails were sampled for a time period of 10–15 minutes [30], at one time and place in each of the 8 sampling sites [28, 31]. The snail collecting techniques used for this study included hand picking and scoop technique [30]. All freshwater snails in a 1-square meter quadrant were collected, placed into plastic bags and labeled by study site and time period. Snails were brought back to the Tropical Disease Research Laboratory, Khon Kaen University and identified based on their morphological characteristics [32, 33]. Particular attention was given to *Bithynia siamensis goniomphalos* (*Bsg*), the *Bithynia* subspecies endemic to the northeastern region of Thailand [33] and host for *Ov*. *Bithynia* snails were distributed into plastic containers with a maximum of 5 snails per cup containing 5 mL of dechlorinated water. These containers were placed under 25 Watt light bulbs for 2 hours for cercarial shedding. The cercariae shed from *Bsg* snails were observed under a stereomicroscope and identified based on their morphological characteristics and distinctive movement as described by Schell [34] and Kaewkes [35]. The *Ov* cercaria body and tail are 154 x 75 μm and 392 x 26 μm, respectively; they are covered in a brownish colored pigment and have two eyespots, oral sucker, pharynx and undeveloped ventral sucker [35]. When observed through a microscope, their morphology combined with their spinning, jerking, floating then sinking motion make them easily identifiable to trained parasitologists and field technicians. In the case of an infected snail cup, snails were separated further into individual cups to identify the infected snail. Once the infected snails were identified a 50 μL drop of water from the cups holding infected individuals was placed on a glass slide and the number of cercariae in that drop was counted allowing for a quantification of cercaria infection intensity.

### Fish sampling and metacercariae sedimentation

The gillnet fishing technique was utilized for this study since it is the fishing method employed by northeastern Lao-Thai fishermen for catching cyprinid fish. Twelve gillnets were custom prepared with a net mesh size of 20 mm, a net length of 5 m, and a net width of 1 m. Gillnets were dropped in all 12 sampling sites between 1:00 p.m. to 4:00 p.m. and picked up the next morning from 6:00 a.m. to 9:00 a.m. All freshwater fish caught in the nets were put into plastic bags, labeled, packed in iceboxes and transported back to the Tropical Disease Research Laboratory, Khon Kaen University. The iceboxes were put in a walk-in cool room (4°C). Approximately 3 days after collection, fish were measured, weighed, and identified according to their morphological characteristics using FishBase [36] and confirmed by experts from the Khon Kaen Department of Fisheries. The fish samples were labeled, separated and digested one-by-one using the Pepsin-HCl Digestion Method, described in Sohn [37]. *Ov* metacercariae were identified morphologically under stereoscopic microscope [35, 37]. *Ov* metacercariae are identifiable by a double-layered thick cyst wall, oval shape and approximate size of 201 x 167 μm in its encysted stage [35]. At this stage and under room temperature, it is possible to see them moving vigorously with a discernible black excretory bladder, oral sucker and ventral sucker.

### Data analysis

#### Environmental data analysis and site characterization

Cluster analysis (CA) and principal component analysis (PCA) were used to characterize the sampling sites and to group them based on their environmental characteristics. CA classifies objects into groups that exhibit high within-cluster homogeneity and between clusters heterogeneity. We implemented the CA using the Hierarchical agglomerative clustering method, which provides similarity relationships between one sample and the entire data set. The Ward’s method was used to normalize the data set using squared Euclidean distances as a measure of similarity and the pvclust() function in the pvclust package provided p-values for cluster similarity significance based on multi-scale bootstrap resampling. In addition to the CA, we used a PCA to identify the water parameters that contributed to the differences/similarities that have led to the clustering patterns revealed by the CA. The number of significant principal components (PCs) was determined based on both scree plot and eigenvalue-one criterion. The eigenvalue-one criterion indicates that PCs with eigenvalues greater than one are regarded as significant when the correlation matrix is used in the analysis. We selected the highest PC loadings (> 0.4) from the projections of normalized water parameter data. Heavy loadings (>0.4) help identify which original variables contribute to the construction of a given component and therefore allow us to determine a synthetic dominant trend for each component.

#### Relationships between site clusters and *Ov* intermediate host abundance, species diversity and prevalence of infection

We computed a generalized linear mixed model (GLMM) to assess the influence of the independent variables “seasonality” (fixed effect), “clusters-specific combinations of environmental parameters” (fixed effect) as well as “single sampling sites” (random effect) on the following response variables: “*Bsg* and cyprinid fish relative abundance” (two variables), “snail and fish species diversity” (two variables), as well as “*Ov* infection rates in *Bsg* and cyprinid fish” (two variables). When relevant, we also computed general models assessing the influence of specific environmental parameters (e.g. salinity) on the responses variables.

*Bsg* and cyprinid fish relative abundance were calculated as the proportion of *Bsg* and cyprinid fish individuals relative to individuals from other snail and fish species. Diversity of snails and fish per site and sampling event (time) was estimated using the Shannon diversity index (H) for which the proportion of species *i* relative to the total number of species (*p*_*i*_) is calculated, and then multiplied by the natural logarithm of this proportion (ln*p*_*i*_). The resulting product is summed across species, and multiplied by -1 (S1 and S2 Tables). Due to the fact that our data set showed low infection rates (zero-inflated data), we computed our models using the logarithm as the (canonical) link function, and the Poisson distribution function as the assumed probability distribution of the responses.

From the information we gained through CA, PCA and GLMM analyses we created maps of Lawa Lake illustrating the spatial and seasonal variability of significant water parameters in relation to snails and fish abundance as well as infection using the Universal Kriging interpolation method [38]. Our data set was fit to a spherical variogram model using inferences that were based on restricted maximum likelihood [39, 40]. The best fitting model and variogram parameters (i.e. sill, range, and nugget) were selected uniquely for each data set by comparing restricted log-likelihood values. Normality was assumed throughout, as initial computation with log-normality assumptions did not yield better interpolations.

All statistical analyses were performed and graphical output created using R version 3.0.1 [41] with packages: graphics [41], cluster [42], pvclust [43], ggplot2 [44], grid [41], devtools [45], factoextra [46], plyr [47], gridExtra [48], ade4 [49], scales [50], vegan [51], FactoMineR [52], Matrix [53], lme4 [54].

## Results

### Environmental data analysis and site characterization

#### Cluster Analysis (CA)

**Fig 2:**
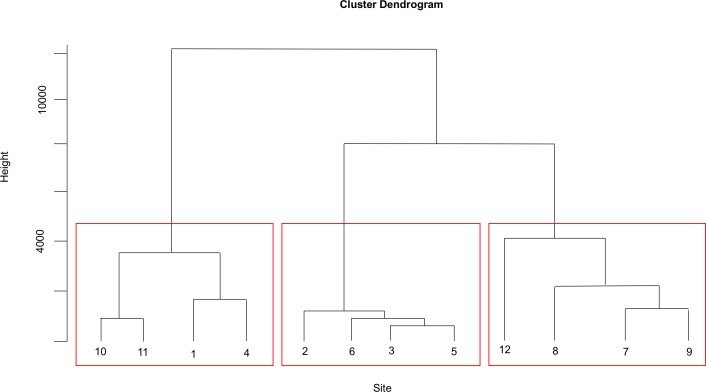
Cluster analysis dendrogram.

Cluster analysis was employed to identify groups of similar sampling sites and to explore spatial heterogeneity of the environmental water quality parameters. It generated a dendrogram, grouping the 12 sites into 3 distinct clusters at (Dlink/Dmax) x 100 < 60 (Fig 2). Cluster 1 included sites, 1, 4, 10 and 11 located along near-shore zones. Cluster 2 included sites 2, 3, 5, and 6, located in the open-water zone. Cluster 3 included sites 7, 8, 9 and 12 located in the southern region of Lawa Lake. The classifications were statistically significant (P<0.05).

#### Principal component analysis (PCA)

The ten water parameters used to examine the water quality of Lawa Lake were pH, Temperature, EC, TDS, Salinity, DO, TCB, FCB, NO_2_-N and Pb (Table 2). PCA was performed on normalized data sets (10 parameters and 12 sampling sites) to reduce the dimensions of the original data sets and to identify latent factors affecting water quality. PCA extracted 5 significant PCs with eigenvalues >1, explaining approximately 86% of the total variance (Table 3).

**Table 2:** Descriptive summary of mean and range of water parameters collected from the 12 study sties in Lawa Lake from Feb. 2013 –Jan. 2014. The highest mean values are marked by asterisks in descending order.

Site		Sal (p.p.t.)	EC (μS/cm)	TDS (mg/L)	pH	Temp (°C)	DO (mg/L)	NO_2_-N (mg/L)	Pb (mg/L)	TCB (MPN/100mL)	FCB (MPN/100mL)
1	Mean	0.3958	785.25	545	6.7783	26.15	1.7167	0.0078	0.0016	3805.8333 *	1459.8333 **
	Range	0.14–0.58	290–1180	191–821	6.06–7.15	21.3–28.4	0.2–3.65	0–0.043	(-0.0032)—0.008	220–16000	11–9200
2	Mean	0.3533	727.25	499	7.9325	26.6917	5.4417 ***	0.0016	0.0013	477	52.5833
	Range	0.14–0.48	298–983	199–680	7.06–9.14	22–29.9	**2.7–8.3**	0–0.006	(-0.0006)—0.006	17–2400	2–540
3	Mean	0.4133	839.5	583	7.05	26.9425	2.2542	0.0013	0.0018	850.5833	208.5833
	Range	0.26–0.55	531–1109	359–772	6.86–7.55	23.7–30.2	0.65–4.7	0–0.005	(-0.0004)—0.01	13–5400	2–1300
4	Mean	0.4108	837	578	6.95	26.775	1.6458	0.0077	0.0088 ***	3255.75	2628.25 ***
	Range	0.19–0.65	402–1324	272–928	6.64–7.44	25–29.1	0.3–2.8	0–0.039	**0.001–0.024**	4–9200	**2–9200**
5	Mean	0.415	853.25	588	7.6508	27.375	4.8625 *	0.0028	0.0019 *	889.4167	11.8333
	Range	0.18–0.68	371–1388	248–978	6.93–8.78	25–30.7	1.05–6.75	0–0.007	(-0.0011)—0.005	14–9200	2–33
6	Mean	0.515	1069.417	735.75	7.4433	27.6083	3.9583	0.0235 *	0.0009	759.5	23.4167
	Range	0.17–0.94	349–2149	233–1370	6.73–8.84	25–30.9	0.85–7.8	0–0.263	(-0.002)—0.006	4–5400	2–94
7	Mean	1.3283	2374	2316 **	7.2242	27.2	4.4583	0.0223	0.0008	1780.5	50.5833
	Range	0.26–2.26	506–4370	578–4790	6.58–7.67	25–29.6	0.85–8.55	0–0.242	(-0.004)—0.002	33–16000	2–170
8	Mean	1.0583	2027.917	1545	6.9733	26.6167	2.1	0.0098	0.0005	619.83	83.4167
	Range	0.34–1.85	689–3650	474–2750	6.73–7.63	20.3–29.5	0.5–6.6	0–0.082	(-0.002)—0.003	49–1600	6–350
9	Mean	1.575 **	2895.167 **	2308 *	7.1792	27.5583	2.7333	0.0423 **	0.0043 **	1325.75	324.9167
	Range	0.43–2.37	892–4580	615–3470	6.58–7.57	25–29.7	0.25–6.1	0–0.486	(-0.0034)-0.016	14–9200	2–3500
10	Mean	1.025	2040.167 *	1497	7.1742	26.5167	3.3125	0.0196	0.0004	5486.6667 ***	680.6667
	Range	0.39–2.08	796–4030	546–3090	6.55–7.72	20–33.3	0–5.85	0–0.168	(-0.0034)—0.003	**110–24000**	2–5400
11	Mean	1.25 *	1768.417	1532	7.1692	27.6667	3.2667	0.1312 ***	0.0012	4458.3333 **	1164.6667 **
	Range	0.22–2.55	461–4260	310–3240	6.26–7.61	23.2–32.4	0.1–6.25	**0–0.664**	(-0.0042)—0.01	110–24000	11–9200
12	Mean	3.6458 ***	5714.75 ***	5369 ***	7.2875	27.6917	5.0875 **	0.0036	0.0015	855.9167	109.1667
	Range	**0.53–14.4**	**1099–24400**	**761–23700**	6.4–9.09	20.1–36.1	1.2–9.55	0–0.011	(0.0059)—0.001	49–5400	2–540

**Table 3:** Principal components (PC) from correlation matrix.

**Eigenvalues (Variances)**										
	**PC1**	**PC2**	**PC3**	**PC4**	**PC5**	**PC6**	**PC7**	**PC8**	**PC9**	**PC10**
	1.783	1.368	1.171	1.034	1.031	0.783	0.663	0.572	0.199	0.161
**Eigenvectors (Principal Component Loadings)**										
	**PC1**	**PC2**	**PC3**	**PC4**	**PC5**	**PC6**	**PC7**	**PC8**	**PC9**	**PC10**
**pH**		0.378	**0.614**	-0.227		-0.102	0.137	0.626		
**Temp**	-0.265	-0.195	0.311	0.331	-0.364	**-0.678**	**0.158**	-0.258		
**EC**	**-0.544**		-0.108			0.115			-0.332	0.742
**TDS**	**-0.543**		-0.117			0.102			-0.469	-0.664
**Sal**	**-0.545**								0.817	
**DO**	-0.174	0.496	0.376	-0.170	0.206	0.129	-0.153	-0.687		
**TCB**		**0.510**	0.261	-0.356	0.261	0.161	0.646	**-0.180**		
**FCB**		**0.519**	0.340	-0.221	0.204	-0.118	-0.710			
**NO**_**2**_**-N**		0.111		0.327	**0.835**	0.396				
**Pb**		-0.142	0.401	**0.725**		0.538				
**Summary of Principal Components**										
	**PC1**	**PC2**	**PC3**	**PC4**	**PC5**	**PC6**	**PC7**	**PC8**	**PC9**	**PC10**
**Cumulative Proportion**	0.318	0.505	0.642	0.749	0.855	0.917	0.961	0.993	0.997	0.100

PC1 expressed heavy loadings (>0.4) on EC (-0.54), TDS (-0.54) and Salinity (-0.55), indicating a ‘salinity’ dominant component since EC and TDS were highly correlated to Salinity. EC, TDS, and Salinity were all negatively correlated with the component, with decreasing Salinity values when PC1 scores increase. PC2 showed heavy loading on TCB (0.51), FCB (0.52) and DO (0.50), therefore named a ‘fecal matter pollution’ component. PC3 reported heavy loadings on pH (0.61) indicating the variation of acidity and alkalinity in the lake depending on wetland features, vegetation and seasonality; PC4 with Pb (0.73) thus influence from heavy metals; and finally PC5 illustrated variation due to NO_2_-N (0.84), indicating a significant impact from sources of nitrogen contamination.

**Fig 3:**
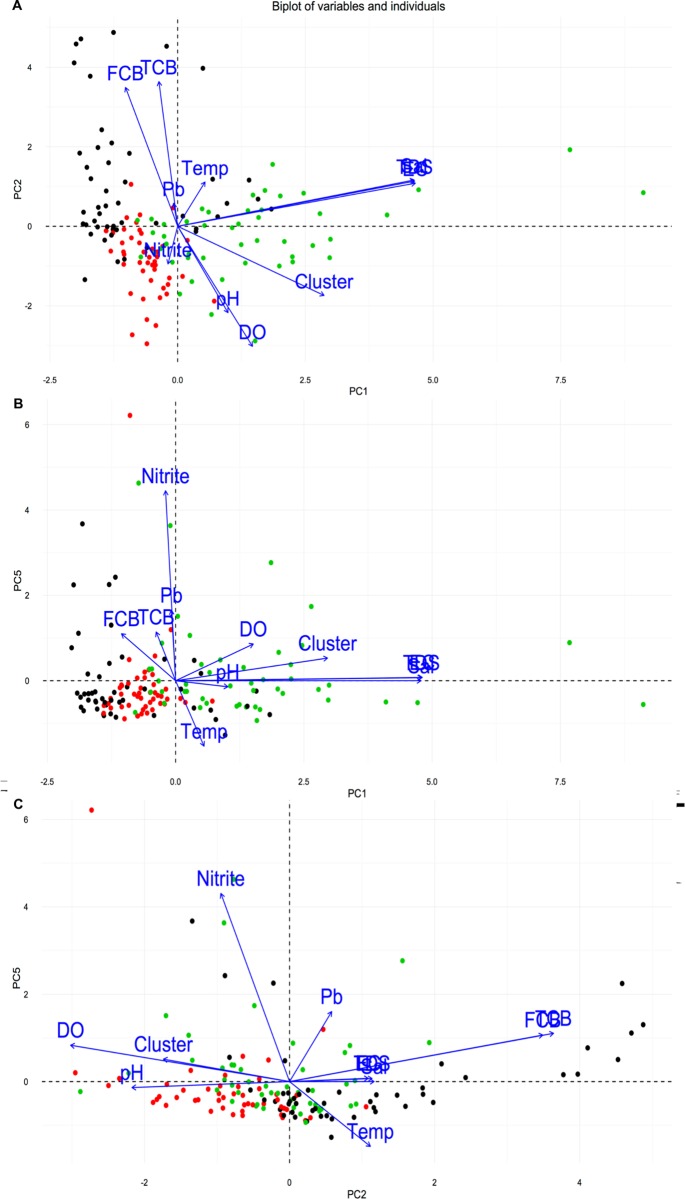
PCA environmental biplot. (A) PC1 –PC2 biplot, (B) PC5 –PC1 biplot, (C) PC2 –PC5 biplot. Sampling sites (dots) and variables (arrows). The green dots represent cluster 3 southern region sites; the black dots represent cluster 1 near shore sites; and the red dots represent cluster 2 deeper water sites.

We observed remarkable partitioning by sampling sites and to a lesser extent by data collection time along PC1, PC2 and PC5, suggesting a directional influence of salinity, fecal matter, and nitrogen variables (Fig 3). In particular, the sampling sites located in the northernmost region of the lake exhibited higher PC1 scores, hence lower salinity related variable values as compared to sites located in the southern region of the lake, (Fig 3, green dots). Northern region sites were also mostly found associated with positive values along PC5, which suggested that sites 1–6 present lower nitrogen values as compared to site 7–12. PC2, a ‘fecal matter’ dominated component indicated geographic variability in fecal matter contamination at the sites nearest to the shore (black dots). Sites 11 and 12 in the southern region of the lake were remarkable for their high salinity and nitrogen values.

PCA demonstrated similar results for sampling sites as the CA. The 3 clusters of sampling sites occupied different ordination space composed by PC1 and PC2. The Water quality of sites 1, 4, 10 and 11 (cluster 1) were mostly negatively correlated with Salinity (-0.5) and NO_2_-N (-0.5) and positively correlated with FCB (0.53). Water quality of sites 2, 3, 5 and 6 (cluster 2) were also negatively correlated with Salinity, TDS and EC (-0.5) as well as demonstrated a strong positive relationship with pH (0.64) and DO (0.59). And lastly, sites 12 and 7, 8, 9 (cluster 3) presented significant negative relationships with Salinity (-0.5), NO_2_-N (-0.54), and FCB (-0.57). Cluster 3 also showed variation and strong negative relationship to pH (-0.61), Temperature (-0.51), and DO (-0.51), opposite to the loading results of cluster 2 that manifested positive relationships with pH and DO.

Lawa Lake is largely utilized as a source of agricultural irrigation and the sites in the southern region reported NO_2_-N levels that exceeded 0.06 mg/L, the irrigation water quality limit recommended by the Food and Agriculture Organization (FAO) [55]. Generally, fecal coliform bacteria from ‘offshore sites’ in the northern region of the lake were less than 1,000 MPN/100 mL. However, the nearshore sites 1, 4, 9, 10 and 11 presented higher loads of fecal coliforms with their highest values occurring during the hot-dry into early hot-rainy season months, specifically from March to June: 9,200; 9,200, 3,500; 5,400; 9,200 MPN/100 mL, respectively. The MPN of fecal coliforms in these shallow nearshore sites exceeded the maximum limit of 1000 MPN of fecal coliforms per a 100 mL sample for irrigation water quality established by the WHO [56].

### Relationships between site environmental variation and *Ov* intermediate hosts abundance and diversity

**Fig 4:**
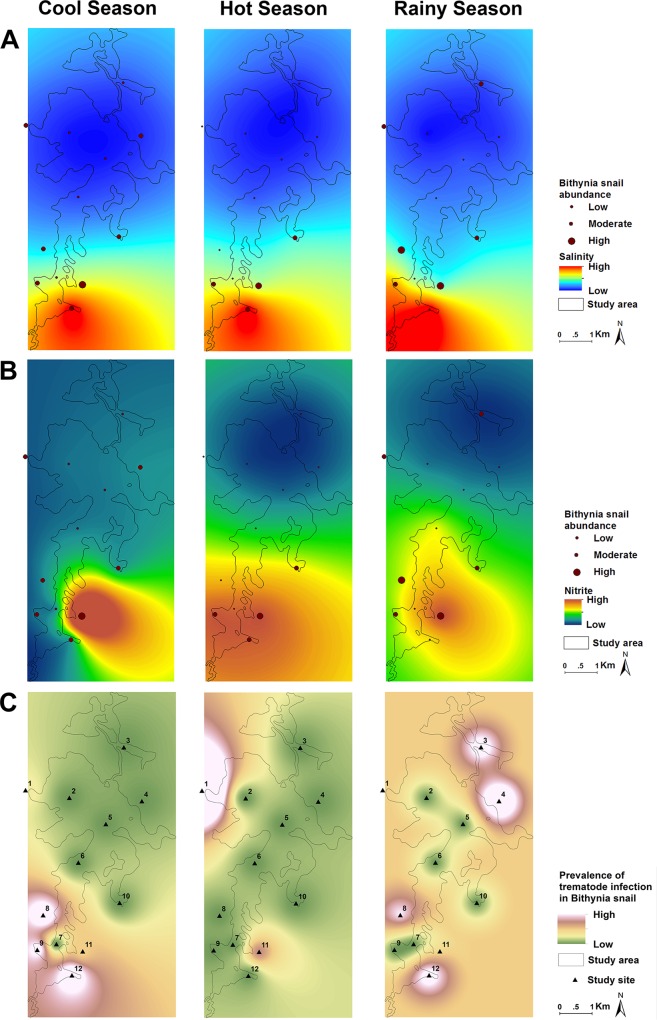
Kriging geographic analysis of the abundance of snails in relation to (A) salinity, (B) nitrite. Panel (C) shows the prevalence of infection of trematode cercariae in *Bsg* snails. Data for the rainy season, cool season and hot season is shown.

There was no significant difference in *Bsg* snail abundance by cluster groups (Table 4). However, *Bsg* snail abundance showed statistically significant differences by season, with the majority of *Bsg* snails collected during the rainy season (*P*<0.001; Table 4). The abundance of *Bsg* snails was highest in October and overall higher in sites located in the southern region of the lake, particularly in site 11, where salinity and NO_2_-N both reported their highest measurements all year. High *Bsg* relative abundance was positively associated with high salinity levels (*P*<0.05, Fig 4A), particularly in site 12 and during February, the end of the cool season. Higher levels of NO_2_-N were positively associated with high *Bsg* relative abundance (*P* = 0.004 and *P* = 0.1, Fig 4B) particularly in site 11 and during the month of April, the country’s hottest month of the year. Snail species diversity showed statistically significant negative association with salinity (*P* = 0.02) but not with site cluster or seasons.

**Table 4:** Generalized linear mixed effects model assessing the influence of seasonality, site clusters and individual sampling sites on the relative abundance, species diversity and *Opisthorchis viverrini* infection rate in *Bsg* snails and cyprinid fish.

Models	Estimate	Std. error	z/t-value	p-value
***Bsg* relative abundance**				
FIXED EFFECTS:				
Site cluster	-0.04545	0.13161	-0.345	0.73
Season	0.24532	0.01933	12.692	<2e-16***
Intercept	3.23409	0.32838	9.849	<2e-16***
RANDOM EFFECTS:		**Variance**	**SD**	
Site		0.2207	0.4698	
**Cyprinid relative abundance**				
FIXED EFFECTS:				
Site cluster	0.36566	0.13043	2.803	0.00506**
Season	0.16062	0.03496	4.595	4.33e-06***
Intercept	0.72971	0.37220	1.961	0.04993*
RANDOM EFFECTS:		**Variance**	**SD**	
Site		0.2701	0.5197	
**Snail species diversity**				
FIXED EFFECTS:				
Site cluster	-0.00385	0.31927	-0.834	0.404
Season	-0.23028	0.17831	-1.291	0.197
Intercept	-0.73466	0.88085	-0.834	0.404
RANDOM EFFECTS:		**Variance**	**SD**	
Site		0.9978	0.9989	
**Fish species diversity**				
FIXED EFFECTS:				
Site cluster	0.005328	0.046672	0.114	0.005328**
Season	0.037845	0.018501	2.046	0.037845*
Intercept	0.862150	0.134421	6.414	0.862150
RANDOM EFFECTS:		**Variance**	**SD**	
Site		0.03429	0.1852	
***Ov* infection in *Bsg* snails**				
FIXED EFFECTS:				
Site cluster	-0.00385	0.31927	-0.012	0.990
Season	-0.23028	0.17831	-1.291	0.197
Intercept	-0.73466	0.88085	-0.834	0.404
RANDOM EFFECTS:		**Variance**	**SD**	
Site		0.9978	0.9989	
***Ov* infection in cyprinid fish**				
FIXED EFFECTS:				
Site cluster	2.0777	0.8179	2.540	0.01107*
Season	-0.2850	0.3124	-0.912	0.36157
Intercept	-9.0148	3.0655	-2.941	0.00327**
RANDOM EFFECTS:		**Variance**	**SD**	
Site		0.4211	0.6489	

**Fig 5:**
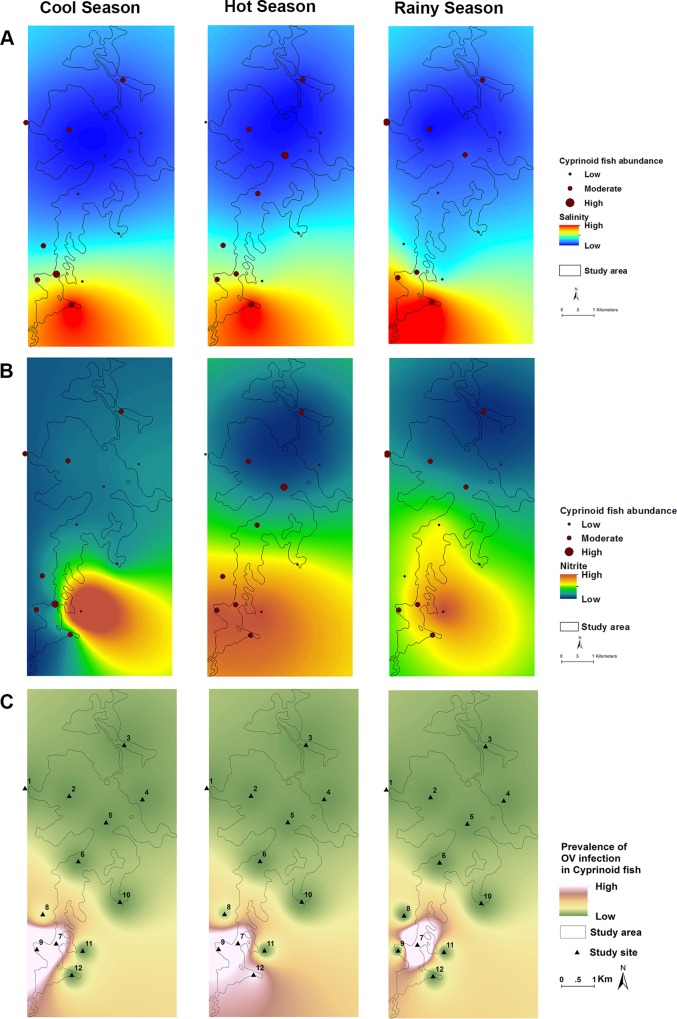
Kriging geographic analysis of the abundance of cyprinid fish in relation to (A) salinity, (B) nitrite. Panel (C) shows the prevalence of *Ov* infection in cyprinid fish. Data for the rainy season, cool season and hot season is shown.

Cyprinid fish were the most abundant family of freshwater fish and were ubiquitously distributed. Cyprinid relative abundance presented statistically significant differences by season (*P*<0.001) with the greatest number of fish being caught during cool season months. Additionally, there were statistically significant differences of cyprinid abundance in the three clusters, (*P* = 0.005; Table 4, Fig 5). The relative abundance of cyprinid fish was greatest in the deep water zone of cluster 2 characterized by strong variations of pH and DO, and the richer-vegetated-southern-region zone of cluster group 3, where *Bsg* abundance, as well as salinity, nitrogen and fecal matter were also at their highest. Fish species diversity was significantly greater in cluster 2 (Table 4) and negatively influenced by salinity and NO_2_-N (both *P <* 0.01).

### Relationships between site environmental variation and infection prevalence

Although *Ov* cercaria infection in *Bsg* snails was nearly undetectable, with only one *Ov* infected snail retrieved during the study period, other trematode infections in *Bsg* snails were detected from 7 out of 8 snail sampling sites. The trematodes infecting *Bsg* snails from Lawa Lake included: *Ov*, amphistome, xiphidiocercariae, parapleurolophocercous, and furcocercous cercariae morphotypes. *Ov* cercaria is the only form that has been specifically identified and named according to its associated trematode species, *Opisthorchis viverrini*. The richness of trematode morphotypes infecting *Bsg* snails as well as the likelihood of infection was highest in sampling site 11 (Table 5), cluster 1 near-shore zone, where we observed the greatest abundance of snails and a significant positive relationship with salinity and NO_2_-N (*P*<0.05; Fig 4C). Despite a singular peak at 0.25% in site 1 during the month of March, *Bsg* trematode infection rate was overall low throughout the year generally ranging from 0.04%–0.09% and consequently seasonality did not significantly influence *Bsg* trematode infection prevalence *(P*>0.05; Table 4).

**Table 5:** A summary table illustrating the sampling site from each month reporting the highest infection rate for snails and fish.

Month	*Bsg* Snail		Cyprinid fish	
	Site reporting highest infection rate	Trematode infection rate (%)	Site reporting highest infection rate	*Ov* infection rate (%)
January	12	0.08	7	0.09
February	8	0.06	9	0.05
March	1	0.25	9 and 12	0.2
April	11	0.05	9	0.12
May	1 and 11	0.06	7	0.16
June	1	0.1	7	0.05
July	4	0.07	7	0.14
August	3	0.05	-	-
September	-	-	-	-
October	-	-	7	0.04
November	9	0.09	8	0.04
December	8	0.04	7	0.05

Fish *Ov* infection prevalence was significantly higher in cluster 3 (Table 4 and 5), the southern region zone that includes sites 7, 8, and 9, exhibiting high fecal matter, salinity and nitrogen pollution, mainly coming from livestock and agriculture. The locations where *Ov* infected fish were collected are close to cluster 1; site 11 in which we found a statistically significant effect of *Bithynia* snail abundance on fish infection rates (*P*<0.05). *Ov* infection in cyprinid fish was not detected in any northern region sampling sites (Fig 5C).

## Discussion

In our study we investigated spatial and seasonal variation in water quality parameters and their influence on *Ov* hosts abundance and infection prevalence. Our objective was to provide novel environmental data to foster a refined understanding of *Ov* transmission dynamics in a highly endemic area and to highlight that knowledge of the ecology of the system needs to be incorporated in interventions planning, including landscape management and local government economic decisions.

### Environmental differences in sampling sites

We observed environmental differences and similarities between the sampling sites mostly in relation to salinity; fecal matter; pH and dissolved oxygen; lead; and nitrogen components, as suggested by the CA and PCA groupings. The observed differences in physicochemical parameters were mostly expressed along a north-south gradient and further discriminated across bathymetric zones. Among the most significant water parameters, we observed higher overall concentrations of salinity and nitrite-nitrogen in sites located in the nearshore and southern region sites, respectively. Two mutually reinforcing sets of determinants thus can potentially explain the patterns of water parameter variation observed: (1) lake hydrological and geomorphological characteristics and (2) human activity varying mostly along the north-south gradient in the region.

The sites contained in the deep-water zone, are located on higher elevations where the lake is wide, open, and deep with moderately strong water flow. Lawa Lake is a dammed reservoir that serves multiple villages for a number of predominantly agrarian services. The dam opening located near Chikokkor Village (cluster 1) facilitates the sustained accumulation of large volumes of water in this area. The deep-water zone (cluster 2) showed typical levels of salinity and nitrite-nitrogen as found in wetland or inland lake ecosystems. These levels were markedly different from those of clusters 1 and 3 where extremely high levels of salinity and nitrogen were measured. The extreme levels of salinity and nitrogen in this area can be explained by differences in water depth associated with marked water stratification, deeper water dilution and homogeneity in contaminant dissemination [57]. Lower elevation sites from cluster 1 and 3, in the southern region zone, where the lake is narrow and shallow, are more severely affected by drought and are characterized by slower water flow, which implies greater water compartmentalization and site-specific biochemical profiles. Coupled with greater intensive agricultural pressure and other human influence, sites from cluster 1 and 3 are more susceptible to contaminant, nutrient and dissolved solid accumulation [57, 58]. The southern region zone also presents a greater concentration of rice paddy fields and a higher overall agrarian pressure, which combined with a lack of human and animal waste monitoring, the unregulated use of agrochemicals and pesticides [59, 60], and general poor irrigation and domestic practices, create a highly contaminated environment, contributing to what others have called a ‘pathogenic landscape’ [13]. In particular, the rural community of Ban Pao, near site 11, has intensified its agricultural practices, mainly rice cultivation and livestock farming [6], with the use of high amounts of agrochemicals and the manipulation of water flow through man-made canals and streams connected to Lawa Lake. The Huoi-jit canal flows through Ban Pao and Ban Nonlamon villages into Lawa Lake and carries with it the waste from the urban settlements of nearby cities such as Ban Phai, an urban area of approximately 20,000 people. Among all sites, site 11 presents particular hydrologic and ecological characteristics, being not only closer to a developed urban area but also more riverine than other sites. These characteristics may have contributed to the unique water quality profile as well as snail and fish community structure in this area as discussed below. Similarly, a plot of land along the shore of Lawa Lake near cluster 3 (sites 7, 8, and 9) is used for the raising of water buffalo, which together with cows, ducks, chickens, pigs and dogs serve as major sources of fecal contamination in the area. Another major source of fecal contamination in the area are humans, who continue to practice open defecation in these aquaculture/rice paddies dominated landscapes, thus contributing to increased fecal coliform contamination and the greater likelihood of snail and fish infection and ultimately parasite transmission [61].

In the specific case of salinity, both historical contingency and current agricultural activities can explain the high levels observed in the southern region zone of the lake. Khon Kaen Province and the Korat Basin, for instance are known to exhibit soils containing high amount of rock salt as a result of pre-historic salt sedimentation in the area [30]. Adding to the inherently high salt content of regional soils, higher agricultural pressure and associated unmanaged irrigation practices in the lower region of the lake induce high rate of salt deposition as a result of intense water evaporation. For example, sugar cane, a cash crop highly prevalent in the area [62], needs about 20,000 m^3^/ha of water per year [63]. As a result, irrigated areas often receive more than 3,000 kg/ha of salt per year and some receive as much as 10,000 kg/ha/year [63].

### Consequences of environmental variation on intermediate host distribution and abundance

Overall, we observed a high relative abundance of *Bsg* snails and cyprinid fish in Lawa Lake, which confirms their potentially important role in sustaining *Ov* transmission in the area. More specifically we observed spatiotemporal patterns in the abundance of both *Bsg* snails and cyprinid fish in cluster 1 and 3, respectively with sites located near the shore and in the southern region of the lake being more prone to *Bsg* and cyprinid fish presence, respectively. These patterns of distribution and abundance may reflect broad scale hydrological influence of the Lawa dam on freshwater snail communities as documented in several recent studies [64]. Dams can alter water flow, sediment flow, and the overall typology of downstream environments [65] causing major ecological alteration in the habitats of humans and aquatic organism diversity [66]. For example, Wu et al [67] looked at how differences in water level and water quality parameter fluctuation impacted the density of schistosoma-transmitting snails, *Oncomelania hupensis*, in Dongting Lake, located near the Three Gorges Dam. They found that low elevation sites saw slight increases of snail density after the completion of the dam, possibly in relation to changes in water temperature, hydrology, vegetation, soil and more indirectly salinity and nitrogen content [67].

Additionally, hydrology and flood plain morphology changes may foster more intensive agricultural practices [68] and other types of development that can lead to water contamination and elevated levels of salinity and nitrogen [69]. For instance, our results suggest that sampling sites located in the southern region of the lake, particularly in the low elevation site 11, which report high levels of salinity and nitrite-nitrogen, are characterized by higher relative abundances of *Bsg* snails and reduced specific diversity of freshwater snail communities. The proximity of site 11 to an urban canal and a developed area with particular hydro-ecological conditions further exemplify the the environment-degradation-pathogen-transmission link as the changes in ecological conditions in these disturbed environments can lead to an unwanted proliferation of disease vectors (sensus lato) such as *Bsg*. The apparent strong influence of these water contaminants on snail community structure and *Bsg* abundance patterns in Lawa Lake indicate species-specific differences in behavior or physiology in snail ability to cope with increased levels of salinity [70] and nitrogen [24]. Previous studies have indicated that while *Bsg* is not found in highly saline freshwater environments, the species prefers water with some saline content (0.05 and 22.11 parts per thousand) over low salinity freshwater [30]; providing further evidence of the possible mediating influence of salinity in freshwater snail communities in the region. Similarly, high levels of nitrogen impair the ability of aquatic animals, including snails and fish, to survive, grow and reproduce and ultimately disrupt freshwater ecological functioning [71]. While no studies have investigated formally the influence of nitrate-nitrogen on *Bsg* survival, elevated nitrate levels in water environments are a major cause of deleterious reproduction and behavior effects for the invasive aquatic snail, *Potamopyrgus antipodarum* [24].

The higher relative abundance of *Bsg* snails and the overall reduced species diversity in lower sites and the implication of high levels of nitrite-nitrogen and salinity in driving this pattern suggest that water contaminants may modulate local freshwater snail community structure (i.e. species richness and abundance) through environment-mediated and species-specific physiological tolerance processes [72]. While this line of interpretation needs further investigation, several recent studies confirm *Bsg* snails as a ‘weedy species’, characterized by high proliferation potential in disturbed/transient environments [73].

The influence of water contamination on cyprinid fish distribution, abundance and community structure is less remarkable than for snails, possibly because of the fish ability to move to or select for favorable habitat across larger distances. Nevertheless, we noticed higher relative abundance of cyprinid fish in sites located in the southern region of the lake particularly in the sites located close to the shore in densely vegetated areas. While further research is needed, this observation may also indicate that some cyprinid species may be better adapted to the ecological situations found in contaminated sites than non-cyprinid species, which may further explain their ubiquitous distribution as a dominant fish family in the area and more generally in SEA [74].

### Consequences on infection and transmission dynamics

Our results suggest that environmental contamination strongly influences patterns of hosts' abundance in a way that sets the stage for increased transmission risks. Indeed we observed higher *Ov* infection prevalence in areas of the lake where high nitrite-nitrogen and salinity levels as well as higher *Bsg* snails and cyprinid fish abundance were found (Table 5). We also found higher *Ov* infection prevalence in fish in water that was contaminated by fecal coliforms, which from the parasite perspective is a medium through which eggs can encounter its first intermediate host. In our study, we used fecal coliform contamination as a proxy for assessing the likelihood of *Ov* presence in the environment. High concentrations of fecal indicators were detected from near shore sites where the boats of fishermen and local huts for shelter were observed. Open defecation is the most direct avenue for *Ov* egg release in the environment in rural areas of northeastern Thailand and Lao PDR where rice farmers spend the majority of their time in the rice fields especially at the onset of the rainy season. The combination of physical presence in the field imposed by the farming practice and low awareness of transmission risks is an incentive for open defecation and creates an ideal transmission arena [75]. While the origin (i.e. human or domestic/wild animals) of the fecal indicators measured in our study cannot be ascertained, it is known that farmers in both northeastern Thailand and Lao PDR tend to prefer open defecation as it can be done immediately in the field and also provides natural fertilizer, a sort of win-win situation, which suggest that human waste is likely to be found diluted in the local water [75]. Echoing [76] further research is needed to clarify the quantitative (how much) and qualitative (what type) relationships between fecal coliforms and egg presence in the environment and improve our ability to use fecal coliforms as a proxy for transmission risks in endemic wetlands.

Despite the high likelihood of *Ov* egg presence in the environment as suggested by high fecal coliform concentrations, and the high human infection prevalence in human communities, particularly around the lower region of the lake (Table 1), we did not find high and regular infection prevalence in *Bsg* snails. Most of the studies surveying *Ov* infection prevalence in snails in endemic areas either fail to find infection or document extremely low infection prevalence typically averaging 0.11% [74, 77]. We believe that the low prevalence usually documented in the literature and the near absence of snail infection reported in our study reflect either 1) the biological processes underlying *Ov* development in the snails and/or 2) the limitation in our ability to detect *Ov* infection in snails through shedding procedures. For instance, infected snails can shed hundreds of cercaria daily [78] and accordingly it is assumed that one infected snail is enough to trigger infection prevalence in fish. It is thus relatively easy to imagine how unlikely finding infected snails can be even in highly endemic areas. Additionally, the technical limitations of infection detectability through cercarial shedding are high [79]. Recent evidence indicates that the routinely used cercarial shedding detection method is greatly outcompeted by molecular detection methods, which identify trematode infection in snails with up to 60.1% higher efficacy. The authors of the study suggested that detection failure is most likely due to the immature and covert infections, which can result in delayed and therefore undetectable (at the time of observation) cercarial shedding [80]. Therefore, the near absence of detected infection in our study may not necessarily be indicative of low snail infection prevalence. While this argument is of speculative nature, year-round *Ov* infection evidence found in cyprinid fish from the same area and high chronic *Ov* infection and sustained transmission in neighboring human communities suggest that infection in snails is hidden and/or their prevalence’s lie below our detection capacity [74]. Adding further complexity, water parameters, such as salinity, are known to modulate greatly snail cercariae shedding patterns, cercariae encystment capacity and overall transmission potential [81] and is likely to play a role in increasing or reducing infection prevalence in fish and thus to blur the relationship between snail infection and fish infection prevalence. Further research is needed to clarify the influence of salinity as well as other water contaminants on snail shedding and cercariae transmission potential in laboratory conditions.

### Conclusion and perspectives on disease prevention and control

Our results indicate that in the southern region of the lake, which is characterized by narrow and complex landscape architecture as well as higher agrarian pressure, water contamination resulting in high nitrite-nitrogen and salinity levels are consistently higher than in other areas. We found that elevated nitrogen and salinity levels were associated with higher *Bsg* snail relative abundance, particularly during the rainy season, and that cyprinid fish relative abundance was higher in the southern part of the lake where vegetation is denser. While infection in snails was nearly undetectable, infection in fish was found year-round in the lower region of the lake where fecal coliform levels, snail and cyprinid relative abundance were the highest. Together, our findings suggest that transmission likelihood is higher in the southern region of the lake where high water contamination, likely associated with intensive agrarian practices and larger scale irrigation schemes affect freshwater communities’ structure and create a particularly “pathogenic landscape” [13]. Indeed, human communities around this part of the lake exhibit particularly high infection prevalence.

Our observations and analyses highlight the highly dynamic nature of *Ov* transmission, both spatially and temporally, and strongly identify it as an ecological process modulated by human and environmental factors [14, 82], for instance agriculture intensification which contributes to water contamination. As such, *Ov* transmission needs to be understood in its social-ecological context [83, 84], including not only natural wetland ecology, but also with an appreciation of the need to connect local landscape management strategies and cultural/agrarian practices/politics with regional and global economic decisions. This is particularly important considering the current context of agriculture intensification and livelihood shifts in northeastern Thailand where unregulated agrochemical use and large-scale irrigation systems disturb local wetlands ecological dynamics while seemingly improving regional and national capital.

Interdisciplinary research and transdisciplinary actions are thus needed to improve our understanding of *Ov* transmission dynamics and to assess carefully the direct benefits and potential direct and indirect losses (e.g. benefits of irrigation versus transmission risk) when managing wetlands (e.g. irrigation projects, agriculture intensification planning, etc.) and, in some instances, to reach compromises and agreed tradeoffs between services and beneficiaries at different administrative scales. This holistic approach can ensure that future interventions designed to reduce *Ov* risk incorporate adaptive management strategies utilizing decision-making and ecological risk assessment tools [15], to be more sustainable and better aligned to improve the governance of health systems [85].

## Supporting Information

S1 Table Number of individual freshwater snails collected, per species, site and sampling event (Time). Although only genus names are provided in the table, only one species per genus is generally found. These are from left to right: *Bithynia siamensis goniomphalos*, *Clea helina*, *Filopadulina martensi*, *Indoplanorbis xxx*, *Lymnaea radix auricularia*, *Melanoides tuberculata*, *Pila polita*, *Scabies xxx*, *Tarebia granifera*, and *Trochataia trochoides*. A diversity measure is also given per site and time. We used the Shannon diversity index (H) to estimate diversity. We also provide evenness (E), which refers to how close in numbers at a particular site and time species are.
(DOCX)

S2 Table Number of individual freshwater fish collected, per species, site and sampling event (Time). Species names abbreviations in order from left to right: *Trichogaster trichopterus*, *Hampala dispar*, *Cyclocheilichthys apogon*, *Labiobarbus lineatus*, *Puntius aurotaeniatus*, *Pristolepis fasciata*, *Osteochilus melanopleurus*, *Thynnichthys thynnoides*, *Oxyeleotris marmorata*, *Notopterus notopterus*, *Osteochilus hasseltii*, *Ompok bimaculatus*, *Tetraodon barbatus*, *Nandus oxyhynchus*, *Channa micropeltes*, *Anabus testudineus*, *Henicorhynchus siamensis*, *Arapaima gigas*, *Puntius brevis*, *Macrognathus siamensis*, *Cirrhinus molitorella*, *Channa striata*, *Osteochilus lini*, *Mystus multiradiatus*, *Mystacoleucus atridorsalis*, *Mystacoleucus ectypus*, *Oreochromis niloticus*, *Parambassis siamensis*, *Cirrhinus jullieni*, *Barbonymus gonionotus*, *Cyclocheilichthys repasson*. Species diversity is also given per site and time. We used the Shannon diversity index (H) to estimate diversity. Evenness (E), referring to how close in numbers at a particular site and time species are, is also given. Species belonging to the family Cyprinidae are indicated by *.
(DOCX)
