# Supplementary material for: Seasonal and Spatial Environmental Influence on Opisthorchis viverrini Intermediate Hosts, Abundance, and Distribution: Insights on Transmission Dynamics and Sustainable Control
Source: PLoS Negl Trop Dis. 2016 Nov 23;10(11):e0005121. doi: 10.1371/journal.pntd.0005121 (PMC5120785; doi:10.1371/journal.pntd.0005121)
Supplement: S1 Table — Although only genus names are provided in the table, only one species per genus is generally found. These are from left to right: Bithynia siamensis goniomphalos, Clea helina, Filopadulina martensi, Indoplanorbis xxx, Lymnaea radix auricularia, Melanoides tuberculata, Pila polita, Scabies xxx, Tarebia granifera, and Trochataia trochoides. A diversity measure is also given per site and time. We used the Shannon diversity index (H) to estimate diversity. We also provide evenness (E), which refers to how close in numbers at a particular site and time species are. (DOCX) [file pntd.0005121.s001.docx]

| **Time** | **Site** | ***Bithynia*** | ***Clea*** | ***Filopadulina*** | ***Indoplanorbis*** | ***Lymnaea*** | ***Melanoides*** | ***Pila*** | ***Scabies*** | ***Tarebia*** | ***Trochataia*** | **H** | **E** |  |
| --- | --- | --- | --- | --- | --- | --- | --- | --- | --- | --- | --- | --- | --- | --- |
| 1 | 1 | 25 | 1 | 0 | 0 | 7 | 0 | 0 | 0 | 0 | 0 | 0,65 | 0,28 |  |
| 2 | 1 | 44 | 0 | 1 | 0 | 3 | 3 | 1 | 0 | 1 | 1 | 0,48 | 0,21 |  |
| 3 | 1 | 51 | 0 | 5 | 0 | 0 | 0 | 2 | 0 | 0 | 1 | 0,11 | 0,05 |  |
| 4 | 1 | 4 | 0 | 0 | 0 | 0 | 0 | 1 | 0 | 0 | 0 | 0,36 | 0,16 |  |
| 5 | 1 | 11 | 0 | 10 | 0 | 0 | 2 | 3 | 0 | 0 | 0 | 1,12 | 0,48 |  |
| 6 | 1 | 55 | 1 | 0 | 0 | 1 | 0 | 0 | 0 | 0 | 0 | 0,64 | 0,28 |  |
| 7 | 1 | 10 | 1 | 1 | 0 | 0 | 0 | 0 | 0 | 0 | 0 | 0,57 | 0,25 |  |
| 8 | 1 | 8 | 1 | 1 | 0 | 0 | 0 | 0 | 0 | 0 | 0 | 0,56 | 0,24 |  |
| 9 | 1 | 28 | 0 | 0 | 0 | 0 | 0 | 0 | 0 | 0 | 0 | 0,14 | 0,06 |  |
| 10 | 1 | 10 | 0 | 0 | 0 | 0 | 0 | 0 | 0 | 0 | 0 | 0,36 | 0,16 |  |
| 11 | 1 | 168 | 0 | 0 | 0 | 6 | 0 | 0 | 0 | 0 | 0 | 7,98 | 3,46 |  |
| 12 | 1 | 37 | 0 | 0 | 1 | 3 | 0 | 0 | 0 | 0 | 0 | 0,20 | 0,08 |  |
| 1 | 2 | 43 | 0 | 0 | 0 | 29 | 0 | 0 | 0 | 0 | 0 | 0,23 | 0,10 |  |
| 2 | 2 | 1 | 0 | 0 | 1 | 38 | 0 | 0 | 0 | 0 | 0 | 0,05 | 0,02 |  |
| 3 | 2 | 1 | 3 | 0 | 0 | 0 | 0 | 0 | 0 | 0 | 0 | 0,32 | 0,14 |  |
| 4 | 2 | 1 | 0 | 0 | 0 | 0 | 0 | 0 | 0 | 0 | 0 | 0,11 | 0,05 |  |
| 5 | 2 | 7 | 1 | 1 | 0 | 0 | 0 | 0 | 0 | 0 | 0 | 0,54 | 0,23 |  |
| 6 | 2 | 31 | 0 | 0 | 0 | 1 | 0 | 0 | 0 | 0 | 0 | 0,16 | 0,07 |  |
| 7 | 2 | 13 | 0 | 0 | 0 | 0 | 0 | 0 | 0 | 0 | 0 | 0,37 | 0,16 |  |
| 8 | 2 | 159 | 2 | 0 | 0 | 0 | 0 | 0 | 0 | 0 | 0 | 7,41 | 3,22 |  |
| 9 | 2 | 22 | 0 | 0 | 0 | 0 | 0 | 0 | 0 | 0 | 0 | 0,27 | 0,12 |  |
| 10 | 2 | 5 | 0 | 0 | 0 | 0 | 0 | 0 | 0 | 0 | 0 | 0,29 | 0,12 |  |
| 11 | 2 | 14 | 0 | 0 | 0 | 0 | 0 | 16 | 0 | 0 | 0 | 0,71 | 0,31 |  |
| 12 | 2 | 2 | 0 | 0 | 0 | 0 | 0 | 0 | 0 | 0 | 0 | 0,17 | 0,07 |  |
| 1 | 3 | 36 | 0 | 0 | 0 | 0 | 0 | 0 | 0 | 0 | 0 | 0,09 | 0,04 |  |
| 2 | 3 | 1 | 0 | 0 | 0 | 0 | 0 | 0 | 0 | 0 | 0 | 0,11 | 0,05 |  |
| 3 | 3 | 8 | 0 | 0 | 0 | 0 | 0 | 0 | 0 | 0 | 0 | 0,34 | 0,15 |  |
| 4 | 3 | 13 | 0 | 0 | 0 | 0 | 0 | 2 | 0 | 0 | 0 | 0,54 | 0,23 |  |
| 5 | 3 | 5 | 0 | 0 | 0 | 0 | 0 | 0 | 0 | 0 | 0 | 0,29 | 0,12 |  |
| 6 | 3 | 24 | 0 | 0 | 0 | 0 | 0 | 0 | 0 | 0 | 0 | 0,23 | 0,10 |  |
| 7 | 3 | 23 | 0 | 0 | 0 | 0 | 0 | 0 | 0 | 0 | 0 | 0,25 | 0,11 |  |
| 8 | 3 | 15 | 1 | 0 | 0 | 0 | 0 | 0 | 1 | 0 | 1 | 0,68 | 0,29 |  |
| 9 | 3 | 14 | 0 | 1 | 0 | 1 | 0 | 0 | 0 | 0 | 0 | 0,58 | 0,25 |  |
| 10 | 3 | 21 | 0 | 0 | 0 | 0 | 0 | 0 | 0 | 0 | 0 | 0,29 | 0,12 |  |
| 11 | 3 | 15 | 0 | 0 | 0 | 0 | 0 | 0 | 0 | 0 | 0 | 0,36 | 0,16 |  |
| 12 | 3 | 42 | 0 | 1 | 0 | 0 | 0 | 0 | 0 | 0 | 0 | 0,20 | 0,09 |  |
| 1 | 4 | 73 | 0 | 0 | 0 | 0 | 0 | 0 | 0 | 0 | 0 | 1,76 | 0,76 |  |
| 2 | 4 | 14 | 0 | 0 | 0 | 2 | 0 | 0 | 0 | 0 | 0 | 0,53 | 0,23 |  |
| 3 | 4 | 34 | 1 | 4 | 0 | 0 | 0 | 0 | 0 | 0 | 0 | 0,33 | 0,14 |  |
| 4 | 4 | 1 | 0 | 1 | 0 | 0 | 0 | 1 | 0 | 0 | 0 | 0,32 | 0,14 |  |
| 5 | 4 | 9 | 0 | 0 | 0 | 0 | 0 | 0 | 0 | 0 | 0 | 0,35 | 0,15 |  |
| 6 | 4 | 3 | 0 | 0 | 0 | 1 | 0 | 0 | 0 | 0 | 0 | 0,32 | 0,14 |  |
| 7 | 4 | 22 | 0 | 0 | 0 | 0 | 0 | 0 | 0 | 0 | 0 | 0,27 | 0,12 |  |
| 8 | 4 | 54 | 2 | 0 | 0 | 0 | 0 | 0 | 0 | 0 | 0 | 0,64 | 0,28 |  |
| 9 | 4 | 179 | 0 | 0 | 0 | 0 | 0 | 0 | 0 | 0 | 0 | 9,17 | 3,98 |  |
| 10 | 4 | 5 | 0 | 0 | 0 | 0 | 0 | 0 | 0 | 0 | 0 | 0,29 | 0,12 |  |
| 11 | 4 | 187 | 0 | 0 | 0 | 0 | 0 | 0 | 0 | 0 | 0 | 9,83 | 4,27 |  |
| 12 | 4 | 57 | 0 | 0 | 0 | 0 | 0 | 0 | 0 | 0 | 0 | 0,94 | 0,41 |  |
| 1 | 5 | 60 | 0 | 0 | 0 | 0 | 0 | 2 | 0 | 0 | 0 | 0,92 | 0,40 |  |
| 2 | 5 | 20 | 0 | 0 | 0 | 0 | 0 | 0 | 0 | 0 | 0 | 0,30 | 0,13 |  |
| 3 | 5 | 13 | 0 | 0 | 0 | 0 | 0 | 0 | 0 | 0 | 0 | 0,37 | 0,16 |  |
| 4 | 5 | 10 | 0 | 0 | 0 | 0 | 0 | 0 | 0 | 0 | 0 | 0,36 | 0,16 |  |
| 5 | 5 | 13 | 0 | 2 | 0 | 0 | 0 | 0 | 0 | 0 | 0 | 0,54 | 0,23 |  |
| 6 | 5 | 28 | 2 | 0 | 0 | 0 | 0 | 0 | 0 | 0 | 0 | 0,31 | 0,13 |  |
| 7 | 5 | 52 | 0 | 0 | 0 | 0 | 0 | 0 | 0 | 0 | 0 | 0,72 | 0,31 |  |
| 8 | 5 | 108 | 0 | 0 | 0 | 0 | 42 | 0 | 0 | 0 | 0 | 4,19 | 1,82 |  |
| 9 | 5 | 37 | 2 | 0 | 0 | 0 | 0 | 0 | 0 | 0 | 0 | 0,04 | 0,02 |  |
| 10 | 5 | 5 | 0 | 0 | 0 | 0 | 0 | 0 | 0 | 0 | 0 | 0,29 | 0,12 |  |
| 11 | 5 | 7 | 0 | 0 | 0 | 0 | 0 | 12 | 0 | 0 | 0 | 0,70 | 0,30 |  |
| 12 | 5 | 23 | 0 | 0 | 0 | 0 | 0 | 2 | 0 | 0 | 0 | 0,42 | 0,18 |  |
| 1 | 6 | 79 | 0 | 0 | 0 | 0 | 0 | 0 | 0 | 0 | 0 | 2,09 | 0,91 |  |
| 2 | 6 | 16 | 8 | 0 | 0 | 0 | 0 | 0 | 0 | 0 | 0 | 0,69 | 0,30 |  |
| 3 | 6 | 0 | 2 | 1 | 0 | 0 | 0 | 0 | 9 | 0 | 0 | 0,63 | 0,27 |  |
| 4 | 6 | 0 | 11 | 6 | 0 | 0 | 0 | 1 | 3 | 0 | 1 | 1,11 | 0,48 |  |
| 5 | 6 | 30 | 2 | 0 | 0 | 0 | 0 | 0 | 0 | 0 | 3 | 0,47 | 0,21 |  |
| 6 | 6 | 60 | 2 | 6 | 0 | 0 | 0 | 0 | 0 | 0 | 0 | 0,61 | 0,26 |  |
| 7 | 6 | 18 | 0 | 5 | 0 | 0 | 0 | 0 | 0 | 0 | 0 | 0,62 | 0,27 |  |
| 8 | 6 | 108 | 19 | 8 | 0 | 0 | 0 | 0 | 0 | 0 | 10 | 2,86 | 1,24 |  |
| 9 | 6 | 1 | 0 | 0 | 0 | 0 | 0 | 0 | 0 | 0 | 0 | 0,11 | 0,05 |  |
| 10 | 6 | 32 | 0 | 0 | 0 | 0 | 0 | 0 | 0 | 0 | 0 | 0,03 | 0,01 |  |
| 11 | 6 | 121 | 0 | 0 | 0 | 0 | 0 | 0 | 0 | 0 | 0 | 4,76 | 2,07 |  |
| 12 | 6 | 74 | 0 | 0 | 0 | 0 | 0 | 0 | 0 | 0 | 0 | 1,81 | 0,79 |  |
| 1 | 7 | 89 | 0 | 0 | 0 | 0 | 0 | 0 | 0 | 0 | 0 | 2,68 | 1,16 |  |
| 2 | 7 | 76 | 0 | 0 | 0 | 0 | 9 | 2 | 0 | 0 | 0 | 1,40 | 0,61 |  |
| 3 | 7 | 58 | 2 | 0 | 0 | 2 | 7 | 3 | 0 | 1 | 0 | 0,00 | 0,00 |  |
| 4 | 7 | 180 | 1 | 0 | 0 | 0 | 5 | 3 | 0 | 7 | 0 | 8,31 | 3,61 |  |
| 5 | 7 | 120 | 0 | 0 | 0 | 0 | 6 | 7 | 0 | 0 | 2 | 3,89 | 1,69 |  |
| 6 | 7 | 135 | 0 | 0 | 0 | 0 | 29 | 0 | 0 | 0 | 0 | 5,65 | 2,45 |  |
| 7 | 7 | 127 | 0 | 1 | 0 | 0 | 0 | 0 | 0 | 0 | 0 | 5,08 | 2,21 |  |
| 8 | 7 | 106 | 0 | 0 | 0 | 0 | 42 | 0 | 0 | 0 | 0 | 4,06 | 1,76 |  |
| 9 | 7 | 154 | 0 | 0 | 0 | 0 | 0 | 9 | 0 | 0 | 0 | 6,83 | 2,97 |  |
| 10 | 7 | 54 | 0 | 0 | 0 | 0 | 0 | 0 | 0 | 0 | 0 | 0,81 | 0,35 |  |
| 11 | 7 | 82 | 0 | 0 | 0 | 0 | 0 | 0 | 0 | 0 | 0 | 2,26 | 0,98 |  |
| 12 | 7 | 28 | 0 | 2 | 1 | 2 | 0 | 0 | 0 | 0 | 0 | 0,59 | 0,25 |  |
| 1 | 8 | 89 | 0 | 0 | 0 | 0 | 0 | 0 | 0 | 0 | 0 | 2,68 | 1,16 |  |
| 2 | 8 | 50 | 1 | 0 | 0 | 1 | 1 | 0 | 0 | 0 | 0 | 0,31 | 0,14 |  |
| 3 | 8 | 4 | 0 | 0 | 0 | 0 | 0 | 0 | 0 | 0 | 0 | 0,26 | 0,11 |  |
| 4 | 8 | 4 | 0 | 0 | 0 | 0 | 0 | 0 | 0 | 0 | 0 | 0,26 | 0,11 |  |
| 5 | 8 | 61 | 0 | 0 | 0 | 0 | 0 | 0 | 0 | 0 | 0 | 1,14 | 0,49 |  |
| 6 | 8 | 64 | 0 | 0 | 0 | 0 | 0 | 0 | 0 | 0 | 0 | 1,28 | 0,56 |  |
| 7 | 8 | 34 | 0 | 0 | 0 | 0 | 0 | 0 | 0 | 0 | 0 | 0,03 | 0,01 |  |
| 8 | 8 | 40 | 0 | 0 | 0 | 0 | 0 | 0 | 0 | 0 | 0 | 0,23 | 0,10 |  |
| 9 | 8 | 31 | 0 | 0 | 0 | 0 | 0 | 0 | 0 | 0 | 0 | 0,06 | 0,03 |  |
| 10 | 8 | 6 | 0 | 0 | 0 | 0 | 0 | 0 | 0 | 0 | 0 | 0,31 | 0,13 |  |
| 11 | 8 | 29 | 0 | 0 | 0 | 0 | 0 | 0 | 0 | 0 | 0 | 0,11 | 0,05 |  |
| 12 | 8 | 12 | 0 | 0 | 0 | 0 | 0 | 0 | 0 | 0 | 0 | 0,37 | 0,16 |  |

**S1 Table.** Number of individual freshwater snails collected, per species, site and sampling event (Time). Although only genus names are provided in the table, only one species per genus is generally found. These are from left to right: *Bithynia siamensis goniomphalos*, *Clea* helina, *Filopadulina martensi*, *Indoplanorbis xxx*, *Lymnaea radix auricularia*, *Melanoides tuberculata*, *Pila polita*, *Scabies xxx*, *Tarebia granifera*, and *Trochataia trochoides.* A diversity measure is also given per site and time. We used the Shannon diversity index (H) to estimate diversity. We also provide evenness (E), which refers to how close in numbers at a particular site and time species are.
